# The Conservation of Chloroplast Genome Structure and Improved Resolution of Infrafamilial Relationships of Crassulaceae

**DOI:** 10.3389/fpls.2021.631884

**Published:** 2021-07-01

**Authors:** Hong Chang, Lei Zhang, Huanhuan Xie, Jianquan Liu, Zhenxiang Xi, Xiaoting Xu

**Affiliations:** ^1^Key Laboratory of Bio-Resource and Eco-Environment of Ministry of Education, College of Life Sciences, Sichuan University, Chengdu, China; ^2^State Key Laboratory of Grassland Agro-Ecosystems, College of Life Sciences, Lanzhou University, Lanzhou, China

**Keywords:** Crassulaceae, chloroplast genome, comparative genomics, phylogenomics, infrafamilial relationships, adaptive evolution

## Abstract

Crassulaceae are the largest family in the angiosperm order Saxifragales. Species of this family are characterized by succulent leaves and a unique photosynthetic pathway known as Crassulacean acid metabolism (CAM). Although the inter- and intrageneric relationships have been extensively studied over the last few decades, the infrafamilial relationships of Crassulaceae remain partially obscured. Here, we report nine newly sequenced chloroplast genomes, which comprise several key lineages of Crassulaceae. Our comparative analyses and positive selection analyses of Crassulaceae species indicate that the overall gene organization and function of the chloroplast genome are highly conserved across the family. No positively selected gene was statistically supported in Crassulaceae lineage using likelihood ratio test (LRT) based on branch-site models. Among the three subfamilies of Crassulaceae, our phylogenetic analyses of chloroplast protein-coding genes support Crassuloideae as sister to Kalanchoideae plus Sempervivoideae. Furthermore, within Sempervivoideae, our analyses unambiguously resolved five clades that are successively sister lineages, i.e., Telephium clade, Sempervivum clade, Aeonium clade, Leucosedum clade, and Acre clade. Overall, this study enhances our understanding of the infrafamilial relationships and the conservation of chloroplast genomes within Crassulaceae.

## Introduction

The angiosperm family Crassulaceae, also known as the stonecrop family, belongs to the order Saxifragales and include 34 genera and approximately 1,400 species (APG IV, 2016; Messerschmid et al., 2020), which are predominantly perennial herbs, subshrubs, or shrubs (Thiede and Eggli, 2007). The species of Crassulaceae primarily occur in (semi-)arid and mountainous habitats of the temperate and subtropical areas (van Ham and Hart, 1998), and are distributed worldwide with centers of diversity in Mexico, southern Africa, Macaronesia, and the Himalayas (Mort et al., 2001). Physiologically, Crassulaceae are characterized by the Crassulacean acid metabolism (CAM) photosynthetic pathway (Gontcharova and Gontcharov, 2009), which achieves a higher level of water-use efficiency than either the C_3_ or C_4_ pathway in water-limited environment (Nobel, 1991). A recent study has identified the crown group of Crassulaceae as one of the 30 core diversification shifts across the angiosperm phylogeny (Magallón et al., 2019). This increased diversification rate may be well associated with the CAM pathway that has been recognized as an evolutionary key innovation (Pilon-Smits et al., 1996; Quezada and Gianoli, 2011; Silvestro et al., 2014).

Until now, numerous phylogenetic studies have been performed to evaluate the infrafamilial relationships of Crassulaceae with a broad taxon sampling (e.g., Berger, 1930; van Ham and Hart, 1998; Mort et al., 2001; Mayuzumi and Ohba, 2004; Gontcharova et al., 2006, 2008; Folk et al., 2019; Messerschmid et al., 2020), which have led to the establishment of three subfamilies by Thiede and Eggli (2007), i.e., Crassuloideae, Kalanchoideae, and Sempervivoideae. Although most of these studies are based on one or a few genetic loci, the monophyly of each of three subfamilies *sensu* Thiede and Eggli (2007) is well supported. The inter- and intrageneric relationships within each of three subfamilies have been also extensively studied using a variety of chloroplast and nuclear loci. The smallest subfamily Crassuloideae comprise approximately 200 species in a single genus, *Crassula* L. (Messerschmid et al., 2020), whose intrageneric relationships have been recently addressed by sampling 103 species (Bruyns et al., 2019). The subfamily Kalanchoideae include approximately 240 species in four genera (Smith et al., 2019), i.e., *Adromischus* Lem., *Cotyledon* L., *Kalanchoe*, and *Tylecodon* Toelken, and the inter- and intrageneric relationships have been exclusively assessed in a few separate studies (Gehrig et al., 2001; Mort et al., 2005; Nowell, 2008). The largest and most complex subfamily Sempervivoideae contain 28 genera and over 1,000 species (Thiede and Eggli, 2007), and the inter- and intrageneric relationships have been investigated by a considerable number of studies (e.g., Mes et al., 1997; Jorgensen and Frydenberg, 1999; Mort et al., 2002; Acevedo-Rosas et al., 2004; Fairfield et al., 2004; Mayuzumi and Ohba, 2004; Gontcharova et al., 2006; Carrillo-Reyes et al., 2008, 2009; Yost et al., 2013; Zhang et al., 2014; Klein and Kadereit, 2015; Nikulin et al., 2016; Vázquez-Cotero et al., 2017; de la Cruz-López et al., 2019). These phylogenetic studies have led to the recognition of five tribes within Sempervivoideae by Thiede and Eggli (2007), i.e., Aeonieae (*Aeonium* Webb and Berth., *Aichryson* Webb and Berth., and *Monanthes* Haw.), Sedeae (*Afrovivella* A. Berger, *Dudleya* Britton and Rose, *Echeveria* DC., *Graptopetalum* Rose, *Lenophyllum* Rose, *Pachyphytum* Link et al., *Pistorinia* DC., *Prometheum* H. Ohba, *Rosularia* Stapf, *Sedella* Britton and Rose, *Sedum* L., *Thompsonella* Britton and Rose, and *Villadia* Rose), Semperviveae (*Petrosedum* Grulich and *Sempervivum* L.), Telephieae (*Hylotelephium* H. Ohba, *Kungia* K.T. Fu, *Meterostachys* Nakai, *Orostachys* Fisch., and *Sinocrassula* A. Berger), and Umbiliceae (*Phedimus* Raf., *Pseudosedum* A. Berger, *Rhodiola*, and *Umbilicus* DC.). Furthermore, based on one nuclear (ITS) and three chloroplast markers (*matK*, *rps16*, and *trnL*-*trnF*), a recent phylogenetic study of 298 Crassulaceae species has recovered six major clades of Sempervivoideae, i.e., Telephium clade, Petrosedum clade, Sempervivum clade, Aeonium clade, Leucosedum clade, and Acre clade (Messerschmid et al., 2020). Despite the significant progress made in the tribal and generic circumscription of Crassulaceae, phylogenetic relationships among tribes and major clades remain poorly to moderately supported or sometimes contradicted, especially within Sempervivoideae (Supplementary Figure 1).

Chloroplasts are semi-autonomous replication organelles, originating in endosymbiosis between cyanobacteria and non-photosynthetic host, and play crucial roles in photosynthesis and physiology of plants (Gao et al., 2019a; Huo et al., 2019; Li et al., 2021). The chloroplast genome is mostly a typical quadripartite circular DNA genome comprising a small single-copy (SSC), a large single-copy (LSC), and two inverted repeats (IRs) (Hu et al., 2016; Chang et al., 2020). With the development of next−generation sequencing technology, chloroplast genomes have been proven to be powerful tools for resolving vague relationships of many complicated lineages due to many advantages, such as uniparental inheritance, the relatively conserved structure, low recombination and substitution rate (Goremykin et al., 2003, 2004; Twyford and Ness, 2017; Li et al., 2020a). Although the structure and sequence of plastomes are relatively conservative, variations in structure, size, and evolutionary rates of genes have been found in many studies, which in some cases signify phylogenetical information and adaptation to environment (Chumley et al., 2006; Hu et al., 2015; Piot et al., 2018; Gao et al., 2019a; Gruzdev et al., 2019; Shrestha et al., 2019; Zang et al., 2019).

Here, we sequenced fourteen fully annotated chloroplast genomes including nine species of Crassulaceae and five species of Haloragaceae. By taking advantage of the data already available, we analyzed a total of 33 chloroplast genomes and aimed to (i) assess the structural characteristics of the chloroplast genome in a comparative framework, (ii) improve the resolution of the infrafamilial relationships within Crassulaceae, and (iii) investigate the adaptive evolution by selective pressures analysis of protein-coding genes in Crassulaceae.

## Materials and Methods

### Taxon Sampling, DNA Extraction, and Sequencing

The aim of our taxon sampling was to try to obtain chloroplast genome sequences for at least one representative of each well-supported major clades. The final taxon sampling contained a total of 26 Crassulaceae species (Table 1), representing all three subfamilies *sensu* Thiede and Eggli (2007), and all five tribes *sensu* Thiede and Eggli (2007) or five out of the six major clades *sensu* Messerschmid et al. (2020) within Sempervivoideae. Of these complete chloroplast genomes, nine were newly generated in this study, i.e., *Aeonium arboreum* (L.) Webb and Berthel., *Cotyledon tomentosa* Harv., *Crassula perforata* Thunb., *Graptopetalum amethystinum* (Rose) E. Walther, *Kalanchoe fedtschenkoi* Raym.-Hamet and H. Perrier, *Orostachys fimbriata* (Turcz.) A. Berger, *Pachyphytum compactum* Rose, *Sempervivum tectorum* L., and *Sinocrassula densirosulata* (Praeger) A. Berger. In addition, seven complete chloroplast genomes were included as outgroups, i.e., *Myriophyllum spicatum* L., *Penthorum chinense* L., and five new sequenced chloroplast genomes of *Haloragis aspera* Lindl., *Haloragis erecta* (Murray) Oken, *Glischrocaryon aureum* (Lindl.) Orchard, *Glischrocaryon glandulosum* (Orchard) Christenh. and Byng, *Gonocarpus micranthus* Thunb. These seven species belong to the family Haloragaceae *sensu lato*, which are the closest living relatives of Crassulaceae (Jian et al., 2008).

**TABLE 1:** Structural information of the chloroplast genomes of Crassulaceae and outgroups.

Family	Subfamily	Clade	Species	Genome size	LSC length	SSC length	IR length	GC-content	No. of	GenBank
				(bp)	(bp)	(bp)	(bp)		PCGs	accession no.
Crassulaceae	Crassuloideae		Crassula perforata*	145,737	79,465	16,652	24,810	37.8%	85	MW206794
	Kalanchoideae		Cotyledon tomentosa*	150,049	82,250	16,995	25,402	38.2%	85	MW206793
			Kalanchoe fedtschenkoi*	150,001	82,015	17,012	25,487	37.7%	85	MW206796
			*Kalanchoe tomentosa*	150,757	82,846	17,051	25,430	37.6%	85	MN794319
	Sempervivoideae	Acre	Graptopetalum amethystinum*	150,365	82,009	16,764	25,796	37.9%	84	MW206795
			Pachyphytum compactum*	149,339	81,041	16,750	25,774	37.9%	84	MW206798
			*Sedum emarginatum*	149,188	81,399	16,721	25,534	37.8%	82	MT680404
			*Sedum japonicum*	149,609	81,429	16,636	25,772	37.7%	85	KM281675
			*Sedum lineare*	149,257	80,963	16,648	25,823	37.9%	85	MT755626
			*Sedum plumbizincicola*	149,397	81,598	16,669	25,565	37.7%	85	MN185459
			*Sedum sarmentosum*	150,448	82,212	16,670	25,783	37.7%	85	JX427551
		Aeonium	Aeonium arboreum*	150,986	82,596	16,706	25,842	37.8%	84	MW206792
		Leucosedum	*Rosularia alpestris*	151,288	82,931	16,785	25,786	37.8%	85	MN794333
		Sempervivum	Sempervivum tectorum*	151,182	82,865	16,709	25,804	37.6%	85	MW206799
		Telephium	*Hylotelephium ewersii*	151,699	83,253	16,838	25,804	37.7%	85	MN794014
			Orostachys fimbriata*	151,195	82,792	16,833	25,785	37.8%	84	MW206797
			*Orostachys japonica*	151,419	83,016	16,849	25,777	37.8%	85	MN794320
			*Phedimus aizoon*	151,393	82,868	17,043	25,741	37.7%	85	MN794321
			*Phedimus kamtschaticus*	151,652	83,010	16,688	25,977	37.8%	85	MG680403
			*Rhodiola integrifolia*	151,452	82,915	17,055	25,741	37.8%	85	MN794327
			*Rhodiola ovatisepala*	151,073	82,348	17,093	25,816	37.7%	85	MN794328
			*Rhodiola rosea*	151,348	82,716	17,052	25,790	37.7%	85	MH410216
			*Rhodiola yunnanensis*	151,257	82,561	17,008	25,844	37.8%	85	MN794332
			Sinocrassula densirosulata*	151,773	83,123	16,904	25,873	37.7%	85	MW206800
			*Sinocrassula indica*	151,755	83,159	16,888	25,854	37.7%	85	MN794334
			*Umbilicus rupestris*	150,995	82,681	16,926	25,694	37.6%	85	MN794335
Haloragaceae *s.l.*			*Penthorum chinense*	156,686	86,735	18,399	25,776	37.3%	84	JX436155
			Glischrocaryon aureum*	158,417	87,743	18,718	25,978	37.1%	83	MW971555
			Glischrocaryon glandulosum*	158,146	88,123	18,743	25,640	36.8%	84	MW971556
			Gonocarpus micranthus*	158,655	88,165	19,000	25,745	42.8%	83	MW971559
			Haloragis aspera*	159,395	89,207	18,482	25,853	36.7%	81	MW971557
			Haloragis erecta*	159,414	89,043	18,555	25,908	36.7%	84	MW971558
			*Myriophyllum spicatum*	158,860	88,420	18,814	25,813	36.5%	84	MH191392

*Species with newly sequenced chloroplast genomes are marked with the asterisks.*

*LSC, large single-copy region; SSC, small single-copy region; IR, inverted repeat region; PCG, protein-coding gene.*

The DNA materials of four species (*Haloragis aspera*, *Haloragis erecta*, *Glischrocaryon aureum* and *Glischrocaryon glandulosum*) were provided by DNA Bank of Royal Botanic Gardens, Kew^1^. Fresh leaves of the other ten species were collected from the field and preserved with silica gel. The total genomic DNA was extracted using a modified CTAB method (Allen et al., 2006). For each species, one paired-end library with an insertion size of ∼350 base pairs (bp) was prepared from the total genomic DNA using the NEBNext Ultra II DNA Library Prep Kit for Illumina (New England Biolabs, MA, United States), which was then sequenced using the HiSeq 2500 System (Illumina, Inc., CA, United States) to obtain paired 150-bp reads. Briefly, (i) the genomic DNA was sonicated using the S220 Focused-ultrasonicator (Covaris, MA, United States), (ii) the fragmented DNA was end-repaired, dA-tailed, adapter ligated, and subjected to 10–12 cycles of PCR amplification, and (iii) the quality of each library was assessed using the 2100 Bioanalyzer system (Agilent, CA, United States).

### Chloroplast Genome Assembly and Annotation

The raw Illumina reads were first filtered to remove paired-end reads if either of the reads contained (i) adapter sequences, (ii) more than 10% of N bases, and (iii) more than 50% of bases with a Phred quality score less than five. The filtered reads were then assembled using NOVOPlasty version 2.7.2 (Dierckxsens et al., 2017), and the complete chloroplast genome of *Sedum sarmentosum* Bunge (Dong et al., 2013) was used as the reference genome. These assemblies were manually inspected using Geneious version 11.0.3 (Kearse et al., 2012).

The assembled chloroplast genomes were annotated using Plann version 1.1 (Huang and Cronk, 2015), and the positions of exons and introns were inspected and adjusted using Sequin version 15.50. In addition, the circular maps of the chloroplast genomes were drawn using OGDRAW version 1.2 (Lohse et al., 2013), and all annotated chloroplast genomes were deposited in GenBank (Sayers et al., 2020).

### Comparative Analysis of Chloroplast Genomes

For the 26 chloroplast genomes of Crassulaceae, the complete nucleotide sequences were compared using the glocal alignment algorithm Shuffle-LAGAN (Brudno et al., 2003) as implemented in the program Mvista^2^ (Frazer et al., 2004). Here, *Rhodiola rosea* L. was chosen as the reference to evaluate gene content variation following Zhao et al. (2020).

To better determine whether any specific pattern of structural variation exists at the family level, the chloroplast genome of *Rhodiola rosea* was used as the representative owing to the highly conserved gene content and gene order within Crassulaceae (see section “Results”), and compared with that of *Penthorum chinense* using progressiveMauve (Darling et al., 2010) as implemented in the software package Mauve version 2.3.1 (Darling et al., 2004). Following Firetti et al. (2017), one of the inverted repeat (IR) regions was manually removed prior to the alignment.

To further identify the hypervariable regions, coding and non-coding regions of the 26 chloroplast genomes were first extracted using PhyloSuite version 1.2.1 (Zhang et al., 2020) and aligned separately using MAFFT version 7.427 (Katoh and Standley, 2013) with default parameters. The nucleotide diversity (π) was then estimated separately for coding and non-coding regions using DnaSP version 6.0 (Rozas et al., 2017).

Moreover, since the size variation of the chloroplast genome may be attributed to the expansion or contraction of the IR region, the boundaries between the IR and single-copy regions were identified using IRscope^3^ (Amiryousefi et al., 2018) and manually inspected using Geneious.

### Phylogenetic Analysis

The nucleotide sequences of the chloroplast protein-coding genes were aligned separately using MAFFT and then concatenated into a supermatrix using PhyloSuite. The optimal partitioning scheme and models of DNA sequence evolution were determined using the relaxed hierarchical clustering algorithm (Lanfear et al., 2014) as implemented in PartitionFinder v2.1.1 (Lanfear et al., 2017).

Phylogenetic relationships were inferred for 26 species of Crassulaceae (i.e., the 33-taxon supermatrix) using both Bayesian inference (BI) and maximum likelihood (ML) methods. For the BI method, four parallel Markov chain Monte Carlo (MCMC) runs were performed using MrBayes version 3.2.7 (Ronquist et al., 2012). The supermatrix was partitioned based on the optimal scheme determined by PartitionFinder, and the best-fitting substitution model was specified as prior for each partition with model parameters unlinked across partitions. A total of 1,000,000 generations were run with sampling every 500 generations, and the first 25% of samples were discarded as burn-in. Convergence of runs was assumed when the average standard deviation of split frequencies dropped below 0.01. The best-scoring ML tree was inferred using RAxML version 8.2.11 (Stamatakis, 2014) with the GTRGAMMAX model for each partition, and branch support was assessed using the rapid bootstrap algorithm (Stamatakis et al., 2008) with 1,000 replicates.

In order to test the potential effect of uneven taxon sampling from each of the genera, we further subsampled the 26 species of Crassulaceae down to a single species as the representative of each genus. Phylogenetic relationships were then estimated from the 20-taxon supermatrix as described above.

### Positive Selection Analysis

The likelihood ratio test (LRT) and Bayes empirical Bayes (BEB) based on modified branch-site model (Yang et al., 2005; Zhang et al., 2005; Yang and Dos, 2011) were used to identify positively selected genes. Since the topological structures of phylogenetic trees constructed by ML and BI methods were congruent, ML tree was used to positive selection analysis. The amino acid sequences of the sixty-seven common protein-coding genes were aligned using MAFFT and converted into nucleotide alignments using PAL2NAL version 14 (Suyama et al., 2006). The nucleotide alignments were trimmed to obtain the final alignments for positive selection analysis by trimAL version 1.4 (Capellagutiérrez et al., 2009). The branch-site model was performed by codeml program in PAML version 4.9 (Yang, 2007). The branch-site test of positive selection was run with the ω of foreground lineage fixed to 1 (fix_omega = 1) for the null hypothesis and estimated (fix_omega = 0) for the alternative hypothesis. The LRT values at df = 1 were calculated by Chi Square test in PAML, and genes with *p* < 0.05 were treated as candidate positives. Finally, BEB was used to identify those positively selected codon sites.

## Results

### Characteristics of Chloroplast Genomes in Crassulaceae

**FIGURE 1:**
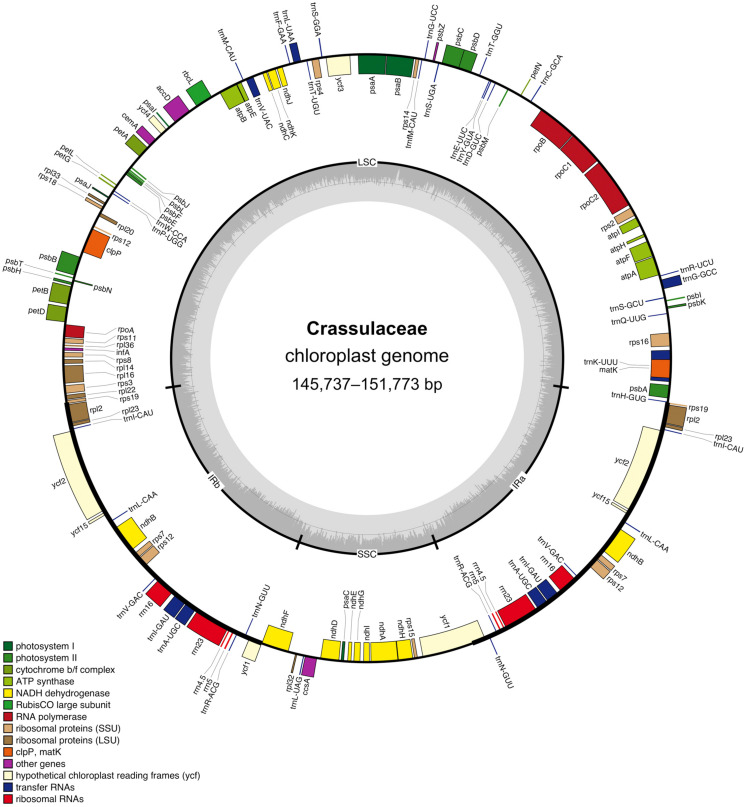
Circular gene map of the Crassulaceae chloroplast genome. The genes labeled inside and outside of the circle are transcribed in clockwise and counterclockwise directions, respectively. The inner circle shows the quadripartite structure, with two IR regions (IRa and IRb) separating the large single-copy (LSC) and small single-copy (SSC) regions. The gray ring marks the GC-content with the inner circle indicating a 50% threshold.

After quality control and pre-processing, at least four gigabases (Gb) of whole-genome sequencing data were obtained for each of the nine species (Table 1). These clean reads were assembled into high-quality chloroplast genomes using a reference-guided approach, and the resulting coverage ranged from 1,115 × (i.e., *Cotyledon tomentosa*) to 12,687 × (i.e., *Kalanchoe fedtschenkoi*). All these newly assembled chloroplast genomes exhibited a typical quadripartite structure, with two IR regions (i.e., IRa and IRb) separating the LSC and SSC regions (Figure 1).

The structure of the chloroplast genome appeared to be largely conserved across the family (Table 1). For each of the 26 Crassulaceae species, the size of the chloroplast genome varied from 145,737 bp (i.e., *Crassula perforata*) to 151,773 bp (i.e., *Sinocrassula densirosulata*), and the overall GC-content ranged from 37.6% (i.e., *Sempervivum tectorum*) to 38.2% (i.e., *Cotyledon tomentosa*). In addition, the total number of annotated genes in each of these chloroplast genomes ranged from 131 (i.e., *Sedum emarginatum* Migo) to 134 (i.e., *Sedum lineare* Thunb.), and all these chloroplast genomes possessed 37 tRNA and four rRNA genes.

**FIGURE 2:**
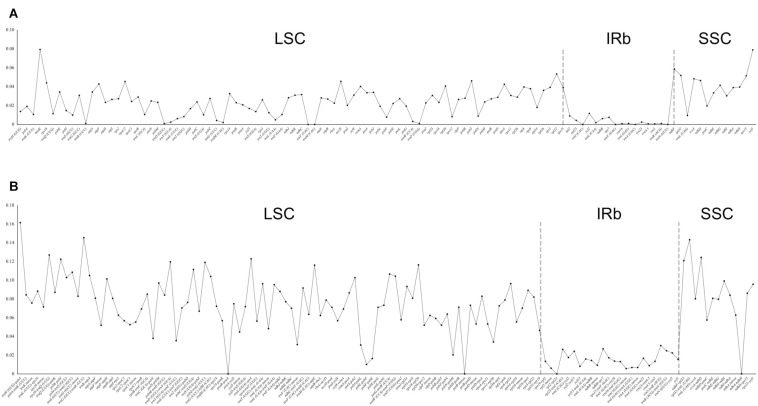
Distribution of nucleotide diversity (π) in coding **(A)** and non-coding **(B)** regions of 26 Crassulaceae chloroplast genomes.

Using *Rhodiola rosea* as the reference, the analysis of mVISTA showed high similarity in gene content and gene order among the 26 chloroplast genomes, and further indicated a fairly high sequence similarity, especially in the coding regions (Supplementary Figure 2). This observation was corroborated by our analysis of nucleotide diversity (Figure 2). The nucleotide diversity in the coding regions ranged from 0 to 0.0794, with an average of 0.0230, which was significantly lower than that in the non-coding regions (0–0.1614, 0.0647; *p*-value < 2.2 × 10^–16^, Welch’s *t*-test). Here, the five coding regions with the highest nucleotide diversity were *matK*, *ycf1*, *ndhF*, *rpl22*, and *rpl32*, and the corresponding non-coding regions were *trnH*-*psbA*, *trnG*-*trnR*, *rpl32*-*trnL*, *rps16*-*trnQ*, and *ccsA*-*ndhD*. Furthermore, no evidence of genomic rearrangement was found in the chloroplast genome of Crassulaceae, when compared with that of *Penthorum chinense* using progressiveMauve (Supplementary Figure 3).

The boundaries of the LSC, SSC, and IR regions were highly consistent within the family, and no obvious expansion or contraction of the IR region was detected in the 26 chloroplast genomes (Supplementary Figure 4). Here, *trnH* was shown to be the first gene in the LSC region at the junction between IRa and LSC (i.e., IRa/LSC). At the other end of the LSC region, the junction LSC/IRb was identified as located within the *rps19* gene, which gave rise to a truncated copy of the *rps19* gene in the IRa region. For both ends of the SSC region, the junctions IRb/SSC and SSC/IRa were found to be located within *ndhF* and *ycf1* gene, respectively. As a consequence, a truncated copy of the *ycf1* gene was retained in the IRb region.

### Phylogenetic Relationships of Crassulaceae

**FIGURE 3:**
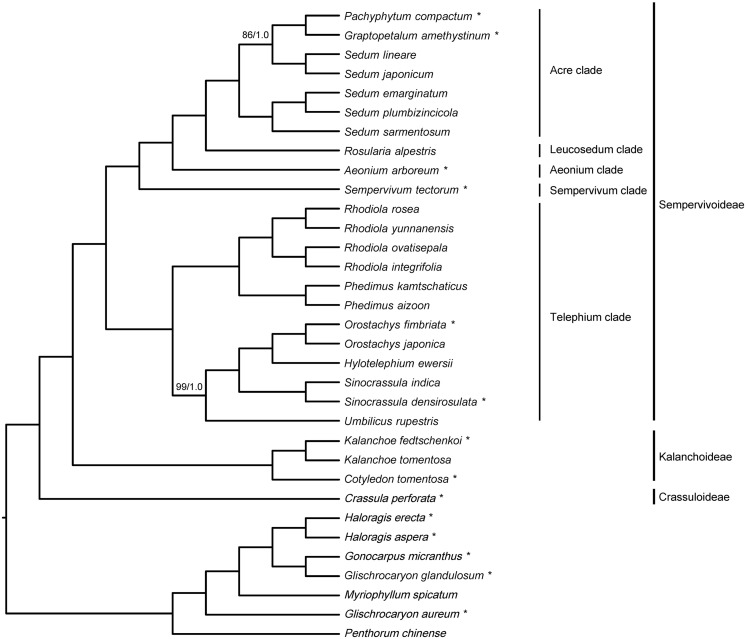
Phylogenetic inference of 33-taxon supermatrix using maximum likelihood (ML) and Bayesian inference (BI) methods. Branch support was assessed using ML bootstrap percentage (BP) and Bayesian posterior probability (PP), and internal branches with less than 100 BP/1.0 PP are indicated with corresponding values. Species with newly sequenced chloroplast genomes are marked with the asterisks, and clade designations are labeled accordingly.

The 33-taxon supermatrix contained a total of 79 genes and 70,905 sites, and the amount of missing data (including gaps and undetermined characters) was 4.5%. Phylogenetic analyses of the 33-taxon supermatrix using ML and BI methods yielded an identical topology (Figure 3), and all relationships were strongly supported by both methods, i.e., ≥85 ML bootstrap percentage (BP) and ≥0.99 Bayesian posterior probability (PP).

All 26 Crassulaceae species formed a monophyletic group, and were divided into three subclades corresponding to the three subfamilies *sensu* Thiede and Eggli (2007), i.e., Crassuloideae, Kalanchoideae, and Sempervivoideae (Figure 3). When rooted with Haloragaceae *s*.*l*., Crassuloideae (represented by *Crassula perforata*) were resolved as sister to Kalanchoideae plus Sempervivoideae. Within Kalanchoideae, the two sampled species of *Kalanchoe* formed a monophyletic group that was sister to *Cotyledon tomentosa*. Within Sempervivoideae, our sampled species fell into five clades, i.e., Acre clade, Aeonium clade, Leucosedum clade, Sempervivum clade, and Telephium clade (Figure 3). Here, the Telephium clade was established as sister to the rest of Sempervivoideae, and further split into two lineages. One lineage comprised two sampled species of *Phedimus* and four sampled species of *Rhodiola*, which formed two reciprocally monophyletic groups, and the other contained the sampled species of *Hylotelephium*, *Orostachys*, *Sinocrassula*, and *Umbilicus*. Importantly, *Umbilicus rupestris* (Salisb.) Dandy was recovered as sister to a clade consisting of *Hylotelephium*, *Orostachys*, and *Sinocrassula*. The Sempervivum clade (represented by *Sempervivum tectorum*) and the Aeonium clade (represented by *Aeonium arboreum*) were placed as successive sister lineages to the Leucosedum clade [represented by *Rosularia alpestris* (Kar. and Kir.) Boriss.] plus the Acre clade. Furthermore, the five sampled species of *Sedum* in the Acre clade were recovered as a paraphyletic group, with *Graptopetalum amethystinum* and *Pachyphytum compactum* nested within them. Importantly, except the non-monophyly of *Sedum*, the same set of intergeneric relationships was recovered from the 20-taxon supermatrix (Supplementary Figure 5), suggesting that our results should be robust to taxon sampling.

### Positive Selection Analysis

A total number of sixty-seven common genes were subjected to positive selection analyses (Table 2). The LRTs with *p*-value > 0.05 suggested that there was no statistical support for positive selection in any genes (Table 2), although the BEB approach identified fourteen genes (*atpB*, *ndhE*, *ndhJ*, *petA*, *psaC*, *psaJ*, *psbB*, *psbD*, *psbN*, *rpoC2*, *rps15*, *rps3*, *rps7*, and *ycf4*) with relatively high posterior probabilities of codon sites.

**TABLE 2:** The positive selection test based on the branch-site model.

Gene name	Null hypothesis		Alternative hypothesis		Significance test	
			
	InL	ω = 1	InL	ω > 1	BEB	*P*-value
*accD*	−5,981.560	1	−5,981.396	999.000		0.566
*atpA*	−5,342.633	1	−5,342.633	3.873		1.000
***atpB***	−4,756.516	1	−4,756.516	1.000	19 Q 0.555; 33 F 0.596; 76 F 0.596; 85 I 0.570; 253 Q 0.585; 269 R 0.585; 307 N 0.592; 392 S 0.600; 424 M 0.593; 450 R 0.580; 458 K 0.599	1.000
*atpE*	−1,313.375	1	−1,313.375	1.000	–	1.000
*atpF*	−2,322.492	1	−2,322.470	436.698	–	0.841
*atpH*	−669.519	1	−670.763	1.000	–	0.115
*atpI*	−2,397.194	1	−2,397.194	1.000	–	1.000
*ccsA*	−4,278.565	1	−4,278.565	3.710	–	1.000
*cemA*	−2,983.494	1	−2,982.704	999.000	–	0.209
*clpP*	−2,044.145	1	−2,043.967	71.380	–	0.549
*infA*	−799.954	1	−799.954	3.322	–	1.000
*ndhA*	−4,381.033	1	−4,381.036	3.345	–	0.920
*ndhB*	−2,843.138	1	−2,843.138	3.042	–	1.000
*ndhC*	−1,206.968	1	−1,206.968	3.034	–	0.997
***ndhE***	−1,161.482	1	−1,160.256	999.000	4 D 0.558; 5 F 0.566; 24 H 0.540; 57 F 0.566; 92 L 0.551	0.117
*ndhG*	−2,177.605	1	−2,177.606	3.061	–	1.000
*ndhH*	−4,525.830	1	−4,525.830	3.179	–	1.000
*ndhI*	−1,904.698	1	−1,904.698	3.538	–	1.000
***ndhJ***	−1,546.588	1	−1,544.996	999.000	1 I 0.566; 14 R 0.576; 33 N 0.560; 51 Q 0.574; 78 F 0.578; 90 F 0.533; 107 N 0.583; 128 R 0.566	0.075
***petA***	−3,388.067	1	−3,388.067	1.000	5 L 0.511; 7 Q 0.504; 32 I 0.511	1.000
*petD*	−1,543.021	1	−1,543.021	1.000	–	1.000
*petL*	−304.819	1	−304.819	3.396	–	1.000
*petN*	−195.153	1	−195.153	3.790	–	1.000
*psaA*	−6,285.558	1	−6,285.558	1.000	–	1.000
*psaB*	−6,370.189	1	−6,370.272	1.000	–	0.689
***psaC***	−650.938	1	−650.673	26.521	53 H 0.553	0.467
***psaJ***	−381.370	1	−381.370	1.000	8 R 0.766; 37 Q 0.759	1.000
*PsbA*	−2,934.741	1	−2,934.728	8.420	–	0.888
***psbB***	−5,011.033	1	−5,011.033	1.000	39 R 0.503; 73 K 0.508; 124 N 0.509; 155 F 0.509; 287 M 0.507; 289 M 0.521; 332 M 0.509; 505 K 0.502	1.000
*psbC*	−4,124.914	1	−4,124.914	1.000	–	1.000
***psbD***	−2,854.422	1	−2,853.539	999.000	13 E 0.571; 159 R 0.518	0.183
*psbE*	−630.873	1	−630.873	1.000	–	1.000
*psbF*	−234.997	1	−234.997	1.000	–	1.000
*psbH*	−766.157	1	−766.157	1.000	–	1.000
*psbI*	−281.572	1	−281.572	3.794	–	1.000
*psbJ*	−283.178	1	−283.178	1.000	–	1.000
*psbK*	−700.844	1	−700.824	609.836	–	0.841
*psbL*	−264.296	1	−264.296	1.000	–	1.000
*psbM*	−329.704	1	−329.704	1.000	–	1.000
***psbN***	−266.750	1	−268.002	1.291	11 F 0.822; 31 P 0.840; 34 Q 0.679	0.114
*psbT*	−332.758	1	−332.758	1.000	–	1.000
*psbZ*	−540.521	1	−540.521	1.000	–	1.000
*rbcL*	−4,364.557	1	−4,364.557	1.379	–	1.000
*rpl14*	−1,092.232	1	−1,092.205	999.000	–	0.823
*rpl16*	−1,532.108	1	−1,532.008	33.646	–	0.655
*rpl2*	−1,729.233	1	−1,729.233	1.000	–	1.000
*rpl20*	−1,410.503	1	−1,410.496	234.313	–	0.903
*rpl23*	−514.597	1	−514.594	5.914	–	0.943
*rpl32*	−768.421	1	−768.421	3.262	–	1.000
*rpl33*	−770.969	1	−770.721	999.000	–	0.480
*rpl36*	−358.642	1	−358.642	6.466	–	1.000
*rpoA*	−4,186.019	1	−4,185.993	999.000	–	0.823
*rpoB*	−12,323.016	1	−12,323.016	2.583	–	1.000
***rpoC2***	−18,495.025	1	−18,494.194	4.364	280 I 0.684; 794 R 0.683; 1,085 L 0.623	0.198
*rps11*	−1,415.271	1	−1,415.250	45.778	–	0.841
*rps12*	−765.506	1	−765.3597	113.044	–	0.590
*rps14*	−989.959	1	−989.959	1.000	–	1.000
***rps15***	−1,369.457	1	−1,369.055	8.032	54 K 0.796	0.370
*rps18*	−849.939	1	−849.939	1.000	–	1.000
*rps19*	−1,186.279	1	−1,186.279	1.979	–	1.000
*rps2*	−2,405.932	1	−2,405.934	1.000	–	1.000
***rps3***	−2,700.278	1	−2,700.278	1.000	74 I 0.505; 88 K 0.620; 89 N 0.504; 105 C 0.550; 180 H 0.590; 199 E 0.584	1.000
*rps4*	−1,960.498	1	−1,960.497	1.000	–	1.000
***rps7***	−786.610	1	−786.345	49.908	12 F 0.914	0.467
*rps8*	−1,693.306	1	−1,693.306	1.000	–	1.000
*ycf3*	−1,302.337	1	−1,302.337	1.000	–	1.000
***ycf4***	−2,054.432	1	−2,054.432	1.000	2 M 0.600; 28 Q 0.646; 33 F 0.573; 54 D 0.595; 151 S 0.520	1.000

*Bold types are genes with positively selected sites identified by BEB with relatively high posterior probabilities.*

## Discussion

In this study, we report nine newly sequenced complete chloroplast genomes of Crassulaceae. Our comparative analyses indicate that the overall gene organization of the chloroplast genome is highly conserved across all 26 Crassulaceae species investigated here. In addition, the results of IRscope analysis reveal no obvious expansion or contraction of the chloroplast IR region. Furthermore, although a recent study has identified a unique 4-kb inversion in the chloroplast genome of the outgroup species *Myriophyllum spicatum* (Liao et al., 2020), our analyses show a high degree of similarity in chloroplast gene order between Crassulaceae and the outgroup species *Penthorum chinense*.

Previous studies have demonstrated that the size variation of the angiosperm chloroplast genome is primarily due to variation in the IR region, intergenic region, and gene copy number (Zheng et al., 2017; Amiryousefi et al., 2018; Bedoya et al., 2019). The results of mVISTA analysis suggest that hypervariable intergenic regions in the LSC region, such as *petA*-*psbJ*, *psbM*-*trnD*, *psbZ*-*trnG*, *rps16*-*trnQ*, and *trnE*-*trnT* (Supplementary Figure 2), contribute most to the chloroplast genome size variation within Crassulaceae. Moreover, phylogenetic studies of Crassulaceae have relied primarily on a limited set of chloroplast markers (e.g., *matK*, *rps16*, and *trnL*-*trnF*). Our comparative analyses, however, have shown that these chloroplast markers appear to be relatively low in nucleotide diversity (Figure 2), which may be partially responsible for the lack of phylogenetic resolution within Sempervivoideae (Mort et al., 2001; Messerschmid et al., 2020). Thus, to achieve better phylogenetic resolution, future studies of Crassulaceae should focus on molecular markers from more variable regions of the chloroplast genome, such as *ccsA*-*ndhD*, *rps16*-*trnQ*, *rpl32*-*trnL*, and *trnH*-*psbA* (Prince, 2015).

With increased taxon sampling of key lineages of Crassulaceae, our phylogenetic analyses of chloroplast genome sequences have substantially improved the phylogenetic resolution, and provided robust inference of the infrafamilial relationships. Among the three subfamilies *sensu* Thiede and Eggli (2007), our phylogenetic analyses have confirmed the monophyly of Kalanchoideae and of Sempervivoideae, and suggested that Crassuloideae are sister to Kalanchoideae plus Sempervivoideae, corroborating previous studies based on DNA sequences and restriction site variation (e.g., van Ham and Hart, 1998; Mort et al., 2001, 2009; Bruyns et al., 2019; Folk et al., 2019). In addition, the sister relationship between Kalanchoideae and Sempervivoideae is supported by two putative morphological synapomorphies, i.e., leaves with a single apical or subapical hydathode and seeds with costate testa (Thiede and Eggli, 2007).

Numerous studies have attempted to identify major lineages within Sempervivoideae (e.g., van Ham and Hart, 1998; Mort et al., 2001; Mayuzumi and Ohba, 2004; Nikulin et al., 2016; Folk et al., 2019; Messerschmid et al., 2020), but relationships among these major lineages remain uncertain (Supplementary Figure 1). Our analyses have revealed five clades that are successively sister lineages, i.e., Telephium clade, Sempervivum clade, Aeonium clade, Leucosedum clade, and Acre clade. These clades correspond to five out of the six major clades *sensu* Messerschmid et al. (2020). Of these six major clades, the lone exception here is the Petrosedum clade *sensu* Messerschmid et al. (2020), for which no chloroplast genome sequence is currently available. The Telephium clade identified here is equivalent to tribes Telephieae plus Umbiliceae *sensu* Thiede and Eggli (2007). Although it was first proposed by van Ham and Hart (1998), the monophyly of the Telephium clade has only recently been confirmed (i.e., ≥85 BP) by phylogenetic analyses of chloroplast genome sequences (Kim and Kim, 2020) and 301 low-copy nuclear genes (Folk et al., 2019). Our results add further evidence to support the recognition of the Telephium clade. In addition, *Umbilicus rupestris* is strongly supported by our phylogenetic analyses as sister to the tribe Telephieae (i.e., our sampled species of *Hylotelephium*, *Orostachys*, and *Sinocrassula*), thus corroborating recent findings (Folk et al., 2019; Kim and Kim, 2020; Zhao et al., 2020) and highlighting the paraphyly of the tribe Umbiliceae (i.e., our sampled species of *Phedimus*, *Rhodiola*, and *Umbilicus*). Furthermore, the Sempervivum and Aeonium clades identified here correspond to tribes Semperviveae and Aeonieae *sensu* Thiede and Eggli (2007), respectively, and the Leucosedum and Acre clades identified here together constitute tribe Sedeae *sensu* Thiede and Eggli (2007).

It has been indicated that the genes with positive selection played key parts in the adaptation to diverse environments (Moseley et al., 2018; Gao et al., 2019b; Li et al., 2020b). However, no positive selection was statistically supported among sixty-seven chloroplast protein-coding genes in sampled Crassulaceae. This result indicated these protein-coding genes are under strong structural and functional constraints. The absence of positive selection in most protein-coding genes of chloroplast genome was also found in *Euonymus* (Li et al., 2021) and *Quercus* (Yin et al., 2018).

In conclusion, by expanding the number of informative molecular characters, we have further improved the resolution of the phylogenetic relationships among major lineages within Crassulaceae, which will facilitate the identification of non-molecular synapomorphies. However, additional sampling of key lineages (e.g., Petrosedum clade) is required to fully resolve the infrafamilial relationships. Furthermore, the boundaries of some of the traditional genera in Crassulaceae remain poorly defined. For example, *Sedum*, the largest genus with approximately 470 species, has been shown to be highly polyphyletic, and its intrageneric relationships remain largely unresolved (Messerschmid et al., 2020). Thus, chloroplast phylogenomics will continue to enhance our understanding of the evolutionary history of Crassulaceae in the future.

## Data Availability Statement

All annotated chloroplast genomes have been deposited in GenBank (https://www.ncbi.nlm.nih.gov/genbank/), and accession numbers are provided in Table 1.

## Author Contributions

JL, ZX, and XX designed research. HC, LZ, and HX performed research and analyzed data. HC, JL, ZX, and XX wrote the manuscript. All authors reviewed and revised the manuscript.

## Conflict of Interest

The authors declare that the research was conducted in the absence of any commercial or financial relationships that could be construed as a potential conflict of interest.

## Footnotes

1 https://dnabank.science.kew.org

2 http://genome.lbl.gov/vista/mvista/submit.shtml

3 https://irscope.shinyapps.io/irapp/
