# Supplementary material for: The Conservation of Chloroplast Genome Structure and Improved Resolution of Infrafamilial Relationships of Crassulaceae
Source: Front Plant Sci. 2021 Jul 1;12:631884. doi: 10.3389/fpls.2021.631884 (PMC8281817; doi:10.3389/fpls.2021.631884)
Supplement: Supplementary file 1 [file Data_Sheet_1.PDF]

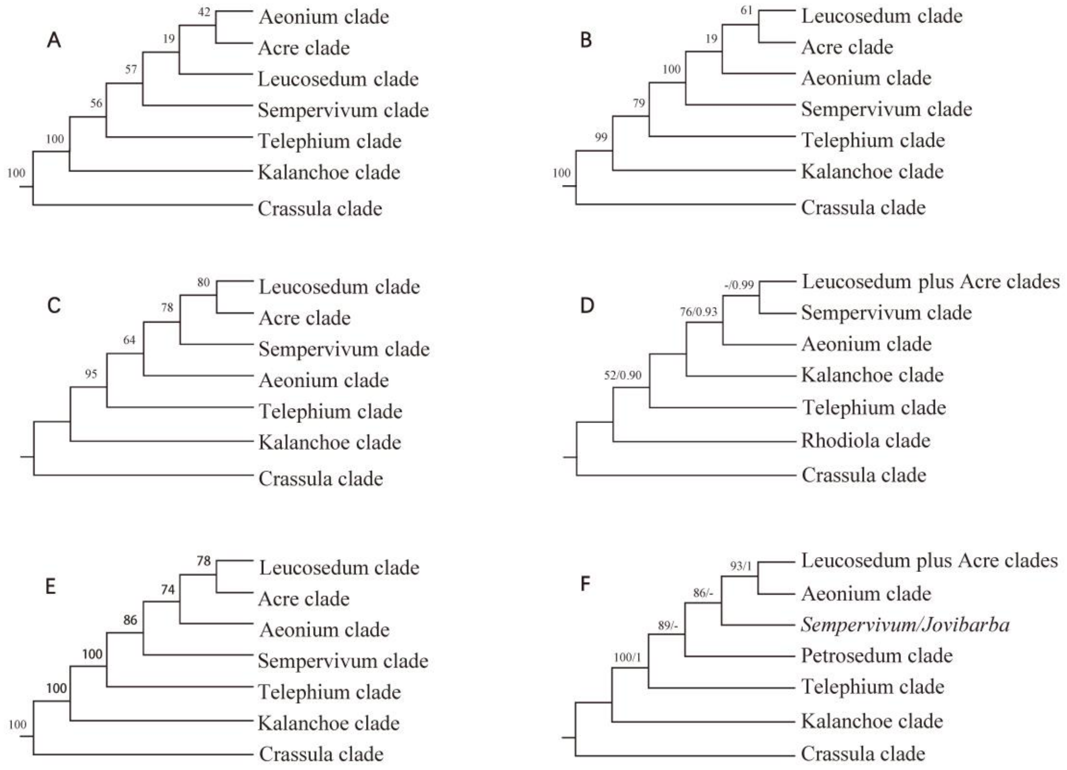

Supplementary Figure 1. Simplified cladograms showing infrafamilial relationships of Crassulaceae inferred in previous molecular phylogenetic studies. **(A)** Parsimony analysis of chloroplast DNA restriction site variation, adapted from Figure 2 of van Ham and ’t Hart (1998). **(B)** Parsimony analysis of chloroplast *matK* sequences, adapted from Figure 2 of Mort et al. (2001). **(C)** Parsimony analysis of chloroplast *trnL-trnF* sequences, adapted from Figure 2 of Mayuzumi and Ohba (2004). **(D)** Maximum likelihood analysis of nuclear ITS sequences, adapted from Figure 4 of Gontcharova et al. (2008). **(E)** ML analyses of 301 protein-coding loci, adapted from Fig. S1 of Folk et al. (2019). **(F)** Maximum likelihood and Bayesian analyses of three chloroplast markers (*matK*, *rps16*, and *trnL-trnF*), adapted from Figure 2 of Messerschmid et al. (2020).

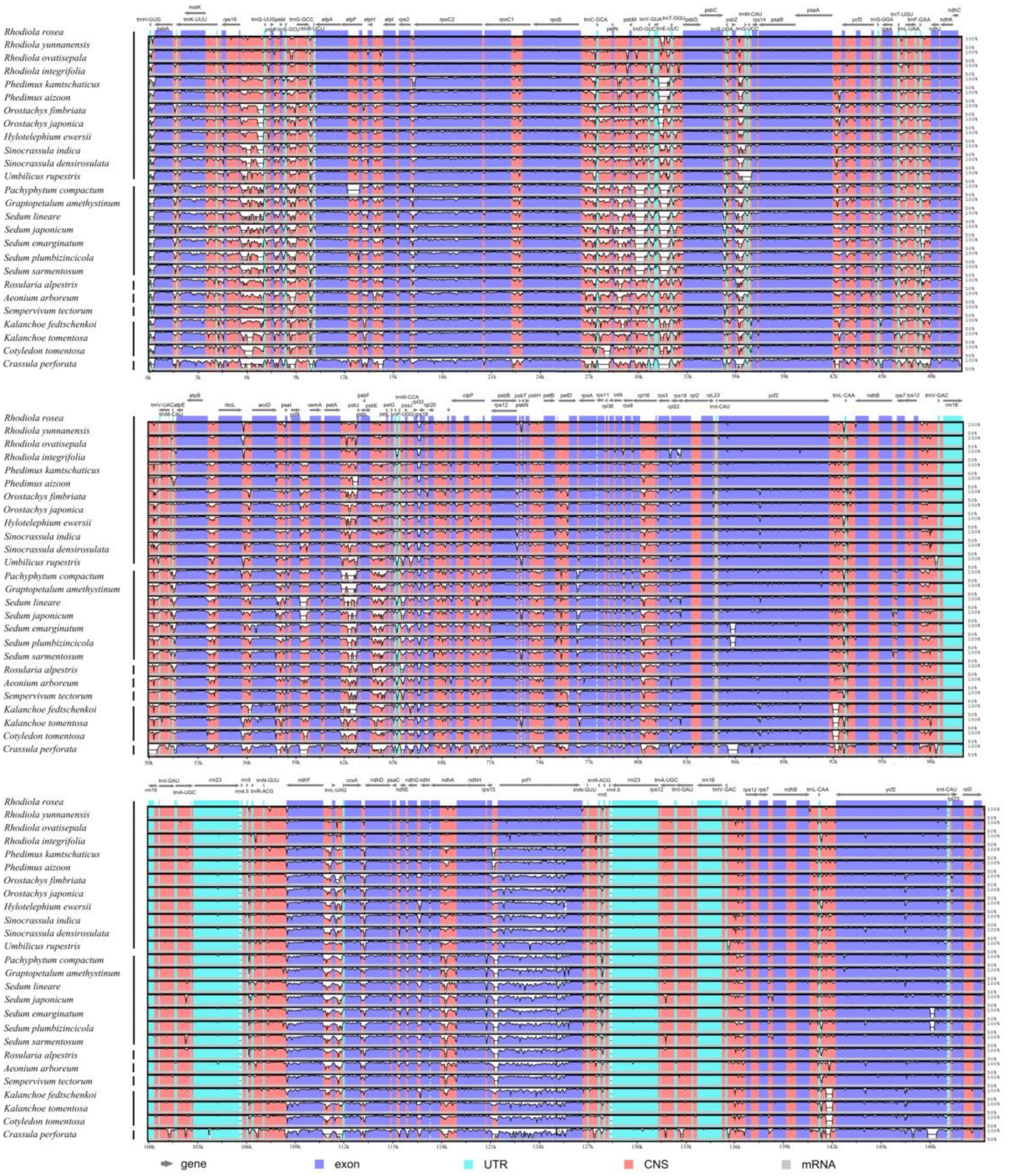

Supplementary Figure 2. mVISTA plot of 26 Crassulaceae chloroplast genomes using Rhodiola rosea as the reference. The horizontal axis represents base positions in the chloroplast genome, while the vertical axis represents the percentage identity (50%–100%). Arrows indicate the direction of transcription of annotated genes in the reference genome.

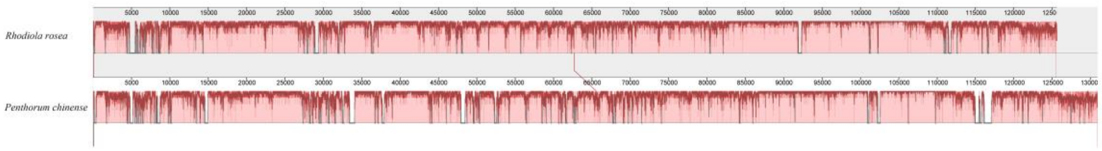

Supplementary Figure 3. Alignment of the chloroplast genomes of *Rhodiola rosea* (Crassulaceae) and *Penthorum chinense* (Haloragaceae *s.l.*) by progressiveMauve. Color bars indicate locally collinear blocks, and connecting lines indicate correspondence of blocks across genomes.

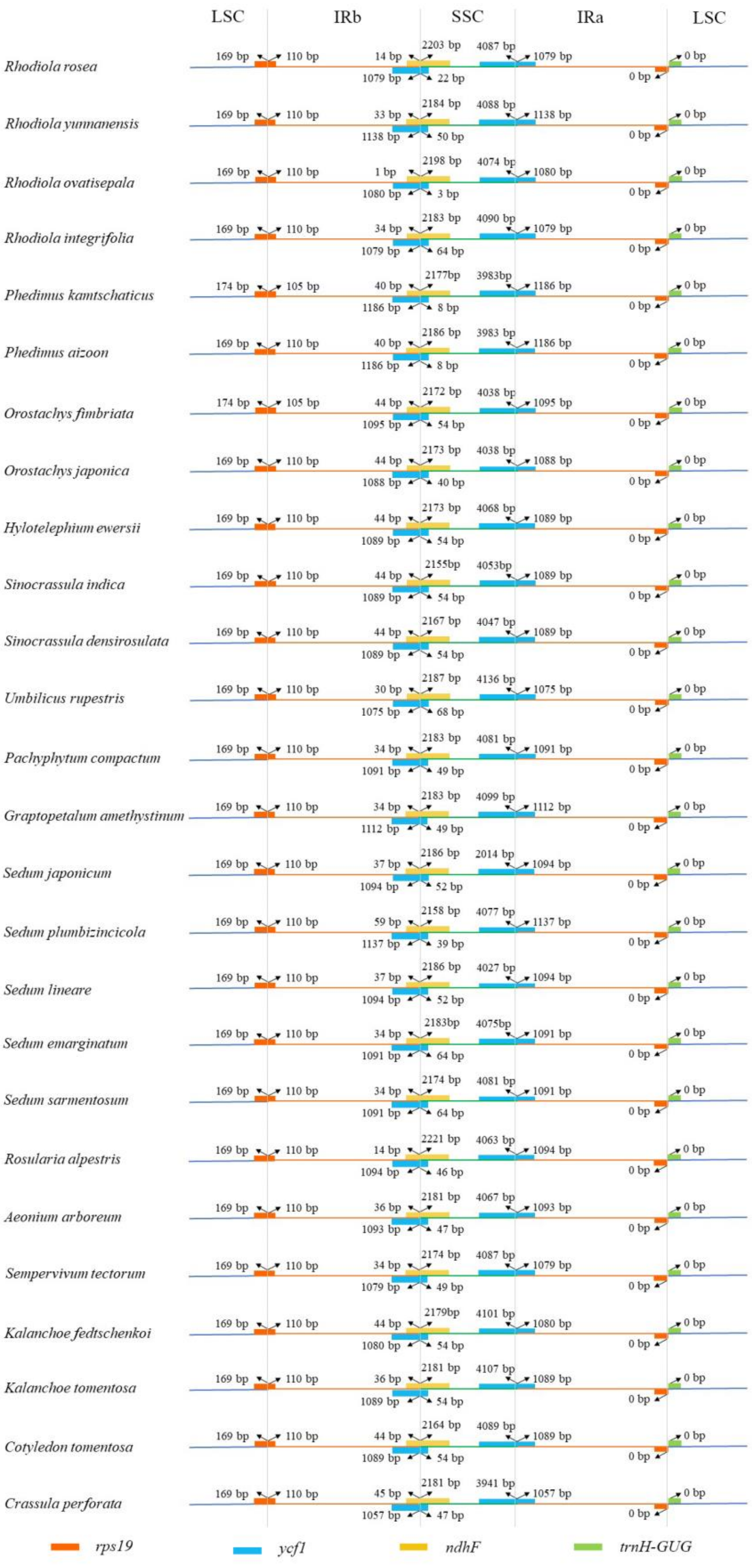

Supplementary Figure 4. Comparison of the boundaries of LSC, SSC, and IR regions among Crassulaceae chloroplast genomes.

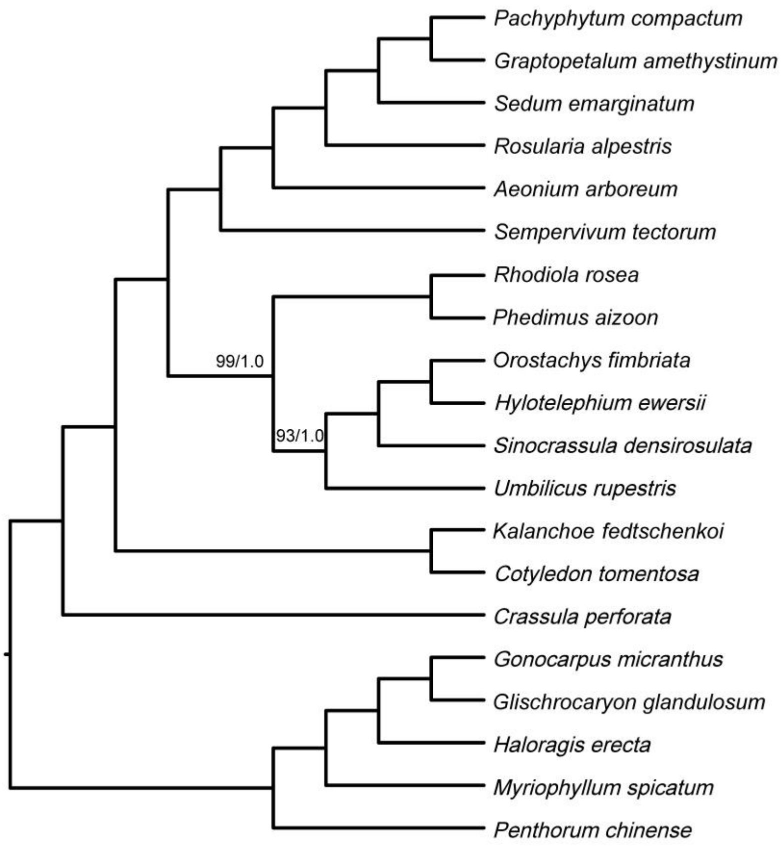

Supplementary Figure 5. Phylogenetic inference of 20-taxon supermatrix using maximum likelihood (ML) and Bayesian inference (BI) methods. Branch support was assessed using ML bootstrap percentage (BP) and Bayesian posterior probability (PP), and internal branches with less than 100 BP/1.0 PP are indicated with corresponding values.
